# Resting State Functional Connectivity Signatures of MRgFUS Vim Thalamotomy in Parkinson's Disease: A Preliminary Study

**DOI:** 10.3389/fneur.2021.786734

**Published:** 2022-01-12

**Authors:** Mario Stanziano, Nico Golfrè Andreasi, Giuseppe Messina, Sara Rinaldo, Sara Palermo, Mattia Verri, Greta Demichelis, Jean Paul Medina, Francesco Ghielmetti, Salvatore Bonvegna, Anna Nigri, Giulia Frazzetta, Ludovico D'Incerti, Giovanni Tringali, Francesco DiMeco, Roberto Eleopra, Maria Grazia Bruzzone

**Affiliations:** ^1^Neuroradiology Unit, Diagnostic and Technology Department, Fondazione Istituto di Ricovero e Cura a Carattere Scientifico (IRCCS) Istituto Neurologico Carlo Besta, Milan, Italy; ^2^Neurosciences Department “Rita Levi Montalcini, ” University of Turin, Turin, Italy; ^3^Parkinson and Movement Disorders Unit, Clinical Neurosciences Department, Fondazione Istituto di Ricovero e Cura a Carattere Scientifico (IRCCS) Istituto Neurologico Carlo Besta, Milan, Italy; ^4^Functional Neurosurgery Unit, Neurosurgery Department, Fondazione Istituto di Ricovero e Cura a Carattere Scientifico (IRCCS) Istituto Neurologico Carlo Besta, Milan, Italy; ^5^European Innovation Partnership on Active and Healthy Ageing, Brussels, Belgium; ^6^Health Department, Fondazione Istituto di Ricovero e Cura a Carattere Scientifico (IRCCS) Istituto Neurologico Carlo Besta, Milan, Italy; ^7^InSightec Ltd., Tirat Carmel, Haifa, Israel; ^8^Neurosurgery Department, Fondazione Istituto di Ricovero e Cura a Carattere Scientifico (IRCCS) Istituto Neurologico Carlo Besta, Milan, Italy; ^9^Pathophysiology and Transplantation Department, University of Milan, Milan, Italy; ^10^Neurological Surgery Department, Johns Hopkins Medical School, Baltimore, MD, United States

**Keywords:** MRgFUS (magnetic resonance-guided focused ultrasound surgery), tremor, Parkinson's disease, ventral intermediate nucleus (VIM), fMRI, resting state functional connectivity

## Abstract

Magnetic Resonance-guided high-intensity Focused Ultrasound (MRgFUS) of the thalamic ventral intermediate nucleus (Vim) for tremor has increasingly gained interest as a new non-invasive alternative to standard neurosurgery. Resting state functional connectivity (rs-FC) correlates of MRgFUS have not been extensively investigated yet. A region of interest (ROI)-to-ROI rs-FC MRI “connectomic” analysis focusing on brain regions relevant for tremor was conducted on 15 tremor-dominant patients with Parkinson's disease who underwent MRgFUS. We tested whether rs-FC between tremor-related areas was modulated by MRgFUS at 1 and 3 months post-operatively, and whether such changes correlated with individual clinical outcomes assessed by the MDS-UPDRS-III sub items for tremor. Significant increase in FC was detected within bilateral primary motor (M1) cortices, as well as between bilateral M1 and crossed primary somatosensory cortices, and also between pallidum and the dentate nucleus of the untreated hemisphere. Correlation between disease duration and FC increase at 3 months was found between the putamen of both cerebral hemispheres and the Lobe VI of both cerebellar hemispheres, as well as between the Lobe VI of untreated cerebellar hemisphere with bilateral supplementary motor area (SMA). Drop-points value of MDS-UPDRS at 3 months correlated with post-treatment decrease in FC, between the anterior cingulate cortex and bilateral SMA, as well as between the Lobe VI of treated cerebellar hemisphere and the interpositus nucleus of untreated cerebellum. Tremor improvement at 3 months, expressed as percentage of intra-subject MDS-UPDRS changes, correlated with FC decrease between bilateral occipital fusiform gyrus and crossed Lobe VI and Vermis VI. Good responders (≥50% of baseline tremor improvement) showed reduced FC between bilateral SMA, between the interpositus nucleus of untreated cerebellum and the Lobe VI of treated cerebellum, as well as between the untreated SMA and the contralateral putamen. Good responders were characterized at baseline by crossed hypoconnectivity between bilateral putamen and M1, as well as between the putamen of the treated hemisphere and the contralateral SMA. We conclude that MRgFUS can effectively modulate brain FC within the tremor network. Such changes are associated with clinical outcome. The shifting mode of integration among the constituents of this network is, therefore, susceptible to external redirection despite the chronic nature of PD.

## Introduction

Patients with Parkinson's disease (PD) have a marked heterogeneity in their clinical features in relation to age of onset, motor presentation/phenotype, neuropsychological profile, and the rate of progression (1, 2). A large body of scientific evidence seems to suggest the existence of four main phenotypes of Parkinson's disease. In addition to the early-onset and late-onset subtypes with rapid disease progression, “motor” subtypes are recognized, particularly the “postural instability and the gait difficulty-dominant,” as well as the “tremor-dominant” subtypes (2). Tremor-dominant PD (TD-PD) is classically characterized by the resting tremor of the limbs, with a common re-emergent component after holding sustained postures (3). Tremor affects the quality of life (4). Patients with TD-PD experience intense embarrassment and difficulties due to their tremor that limit social interactions and frequently interferes with their ability to perform the daily living activities and simple tasks both at home and at work (5). Tremor is primarily managed with medications, but both response to tremor and satisfaction with medical therapy are highly variable (5). Moreover, effective medications can be associated with adverse effects (6–8).

The search for increasingly effective therapies drives to a better understanding of the pathophysiology of the disorder and the possible targets for non-pharmacological treatments, such as surgical lesions or neuromodulation. The actual pathophysiology of this disabling phenomenon is still partially undetermined, and the proposed mechanisms are currently under debate. Altered interactions between the cerebello-thalamo-cortical circuitry and the basal ganglia are thought to contribute to parkinsonian tremor (9, 10). In addition, dopamine depletion in the globus pallidus has been historically associated with the severity of clinical tremor (9). These assumptions are echoed by the recent “*finger-switch-dimmer*” hypothesis (11), for which tremor in PD would be: (i) induced by pathological triggering from the dopamine-depleted basal ganglia; (ii) generated by changes in the oscillatory activity within related thalamic nuclei (i.e., from pallidal to cerebellar thalamic recipients); and (iii) modulated by the cerebellum. Efferent copies of tremorigenic thalamic activity would be transmitted to the cerebral sensorimotor cortex, then it will be fed back into the basal ganglia and also propagated to the subthalamic nucleus through thalamo-cortical, thalamo-striatal, cortico-striatal, and cortico-subthalamic pathways (12, 13). Therefore, PD tremor would be mediated by parallel and only apparently segregated trans-cortical and sub-cortical circuits converging to the thalamus (14).

Even though none of the above described “circuital perspectives” is likely to explain definitively how tremor is generated and propagated in TD-PD and similar disorders, such as essential tremor (15), tremorigenic disorders would appear to share a common dysfunctionally distributed tremor-network centered on the thalamus, specifically on the thalamic ventral intermediate nucleus (Vim) (16). Vim is the cerebellum-recipient nucleus of the thalamus and has traditionally been regarded as the preferred target for neuromodulation or lesional neurosurgery to obtain tremor relief (17, 18). Growing recent evidences have shown that effectiveness of interventional procedures for tremor may be related to the proximity between the actual Vim lesion and the white matter tracts extending through the Vim, namely the dentato-rubro-thalamic tract (DRTT) (19–31). Vim ablation would, therefore, interfere with the tremor-sustaining aberrant circuitry (32).

In recent years, promising results have been published on the thalamotomy of the Vim using Magnetic Resonance-guided high-intensity focused ultrasound (MRgFUS). This is a non-invasive procedure performed under MRI guidance which allows to produce a small lesion (i.e., a focal area of coagulative necrosis induced by heat) at the level of selected target (i.e., the Vim) (33). This procedure represents an interesting therapeutic option for parkinsonian tremor that is not responsive to pharmacological therapy in cases where patients do not want to undergo or have contraindications to invasive procedures, such as deep brain stimulation. Subsequently, this technique is increasingly being employed as both safe and effective symptomatic treatment for medication-resistant, long-lasting, and disabling tremor in patients suffering from TD-PD.

To the best of our knowledge, there are only a few studies describing functional correlates of Vim ablation, and the potential mechanisms of connectivity reorganization over time after lesional procedures on Vim (mainly based on stereotactic radiosurgical thalamotomy) are yet to be recognized. To date, the literature on PD only includes at most 10 patients (34–42).

Here, we used resting state functional MRI (rs-fMRI) to longitudinally explore the dynamics of functional interactions between different nodes of the above-described “*tremor-network”* before and after the MRgFUS Vim ablation in a cohort of patients with TD-PD (“*Main effect”* of MRgFUS treatment). In particular, our goal was to evaluate if the changes in rs-fMRI interactions were transient and limited over time; for example, occurring only at 1 month after treatment mainly due to early postoperative alterations, or if they were still identifiable at 3 months after the complete postoperative oedema reabsorption.

In addition, we examined whether: (i) disease duration was related to changes in intra-subject FC between the areas that are forming part of the tremor-network (“*treatment by disease duration interaction effect”*); (ii) post-MRgFUS Vim lesion's volume at 24 h influenced the FC changes (“*treatment by lesion volume interaction effect”*); (iii) FC changes correlated with clinical improvement at 3 months after MRgFUS (“*treatment by clinical improvement interaction effect”*); (iv) FC changes differed between clinical outcomes (“*treatment by outcome interaction effect”*). Finally, (v) we attempted to retrospectively identify FC features at baseline that might be predictive of different clinical outcomes (“*pretherapeutic functional profiles of outcomes”*).

## Materials and Methods

We prospectively enrolled 60 consecutive patients with idiopathic TD-PD [according to clinical diagnostic criteria for Parkinson's disease of the Movement Disorder Society: (43)], with disabling tremor resistant to medication, who were evaluated at our institution from January 2019 to June 2020. All patients were carefully evaluated by a neurologist expert in movement disorders [RE; NGA; SB] and were considered good candidates for MRgFUS unilateral Vim thalamotomy. In particular, patients were examined in “off” (at least 12-h overnight withdrawal of antiparkinsonian therapy) and “on” conditions (90 min after a levodopa loading dose, approximately equal to 150% of the patients' usual morning dose) by the part-III items of the Movement Disorder Society Unified Parkinson's Disease Rating Scale [MDS-UPDRS: (44)].

Main *inclusion criteria* for MRgFUS were: (i) medication-refractory disabling tremor, defined as “disabling in the main activities of daily life despite of all available oral treatments” and confirmed by “acute levodopa challenge response”; (ii) age > 18 years; and (iii) contraindication for deep brain stimulation (DBS) or patients who refused DBS.

*Exclusion criteria* were as follows: (i) other neurodegenerative diseases than PD; (ii) history of prior stereotactic neurosurgery or DBS; (iii) standard contraindications for MR-imaging or for MRI contrast agent; (iv) patient/s unable to tolerate supine position for long time during treatment (4+ h) or claustrophobia; (v) significant cognitive impairment documented by neuropsychological evaluation (Mini-Mental State Examination ≤ 21); (vi) serious psychiatric pathologies, active drug/alcohol dependency, or prior abuse; (vii) risk factors for bleeding, unstable cardiac status, or medical conditions not allowing anticoagulant/antiplatelet therapy discontinuation; (viii) history of intracranial hemorrhage or stroke within the past 6 months; (ix) history of seizures within the past year; (x) presence of brain tumors; and (xi) a skull density ratio (SDR) (45) ≤ 0.34 as calculated from the head computed tomography screening scan.

### Study Design and Outcome Definition

The rs-fMRI data were acquired at baseline (during the screening stage, not exceeding 4 months before MRgFUS treatment), as well as at 1 month (1-mo) and 3 months (3-mo) postoperatively. Clinical assessment was usually performed on the same day of each MRI examination or, at most, the day before or after. For this study, the final clinical outcome was defined by the score variation of the tremor improvement at 3 months (3-mo) post-treatment, regarding the body side contralateral to the thalamotomy, and calculated as absolute drop value (score at 3-mo minus score at baseline of the sub-items 3.15.a, 3.16.a, 3.17.a, and 3.17.c of the MDS-UPDRS scale in the off-state) (39), and also, as percentage of intra-subject value (% = baseline minus 3-mo/baseline score x 100) (20). We adopted a clinical and an rs-MRI evaluation in an off-drug condition because we were interested in the MRgFUS thalamotomy effect on FC without any pharmacological influence. Considering a quartile ranking on the degree of effectiveness (46), improvements of ≥50% compared to baseline were considered as therapeutic, while those <50% as sub-therapeutic, further defining two outcome subgroups (good vs. poor responders, GR vs. PR).

### MRI Data Acquisition and Processing

Transcranial MRgFUS Vim-thalamotomy was performed by the ExAblate 4000 system (InSightec, Haifa, Israel) installed on a 1.5T MR scanner. Screening and follow-up of fMRI data were acquired on a 3T scanner equipped with a 32-channel coil (Achieva TX, Philips Healthcare BV, Best, NL). High resolution volumetric turbo field echo T1-weighted (TR = 8,200 ms; TE = 3,700 ms; flip angle = 8°; voxel = 1 × 1 × 1 mm) and T2-weighted (TR = 2,500 ms; TE = 2,800 ms; flip angle = 90°; voxel = 0.8 × 0.8 × 0.8 mm) images were acquired. The rs-fMRI acquisition consisted of a repeated gradient-echo planar imaging sequence (TR = 3,000 ms, TE = 30 ms, α = 80°, 2.5 mm isotropic voxel size, matrix size = 90 x 95) providing 47 ascending interleaved images per volume, parallel to the anterior commissure-posterior commissure (AC–PC) line and covering the whole brain.

Importantly, the patient habitual pharmacological treatment for tremor was discontinued at least 12 h before the MRI scanning session.

The rs-fMRI data preprocessing and analysis were performed using the Statistical Parametric Mapping (SPM12, http://www.fil.ion.ucl.ac.uk/spm), and the CONN toolbox (release 19.c) (47) running on MATLAB R2019b (MathWorks, Natick, MA, USA). Scans of patients with right thalamotomy were preliminary flipped so that treatments were all conventionally considered on the left hemisphere. Therefore, we could define a treated side (TS) for the whole study sample, corresponding to the left cerebral hemisphere with contralateral (right) cerebellum, as well as the untreated side (unTS), corresponding to the right cerebral hemisphere with contralateral (left) cerebellum. Functional images were realigned, unwrapped, and slice-time corrected. Gray-matter (GM), white-matter (WM), and cerebrospinal fluid (CSF) were automatically segmented, and the functional data were normalized to the Montreal Neurological Institute (MNI) template. Data were spatially smoothed with a Gaussian kernel set at 6 mm full width at half maximum. The first five principal components from WM and CSF signals, the six motion realignment parameters, and their first-order derivatives, as well as the outlier volumes were detected using the ART-based scrubbing method (48) as implemented in CONN, and were regressed out of the signal. Subjects with excessive head motion in one of the 3 follow-up scans (i.e., ≥50% of volumes detected as outliers) were excluded from further analysis. Accepted data were then band-pass filtered (0.008 to 0.1 Hz) and were linearly detrended.

Resting-state functional connectivity was tested with region-to-region (“*connectomic”*) analysis. Most region-of-interest (ROI) masks were already in the probabilistic Harvard-Oxford (49) and AAL (50) atlases included in the CONN toolbox. We chose ROIs potential relevance for tremor pathogenesis according to existing literature [in particular see (36)]: precentral (PreCG) and postcentral (PostCG) gyrus; supplementary motor area (SMA); paracingulate gyrus (PaCiG); and median anterior cingulate (mAC), covering the most caudal part of pre-supplementary motor area, as well as the cingulate motor areas; inferior and middle frontal gyrus, encompassing the ventral and dorsal premotor cortex (vPMC and dPMC); superior parietal lobe (SPL); temporal-occipital fusiform cortex (TOFusG); and occipital fusiform gyrus (OFusG) [in particular see (36)]; putamen; pallidum; and all cerebellar lobules (Lob), including vermian subdivisions (Ver). Additional ROI in the thalamus (Th) was initially defined based on patient's lesions and then imported in CONN toolbox; in details, individual MRgFUS thalamic lesions were semi-automatically outlined on the 24-h post-treatment volumetric T2-w images (or, when not available, post-contrast T1-w images) using the ITK-Snap software. Only voxels that were rated by two independent expert observers [MS; GD], as belonging to zones I and II of a particular lesion, were included in the final lesion mask, while the surrounding vasogenic oedema (zone III) (33) was excluded. Lesion masks were normalized to MNI in SPM, and then, were averaged across subjects to create a group thalamic ROI, encompassing all the post-operative Vim (TS Th-Vim). The flipped contralateral ROI was set as the unTS Th-Vim. Moreover, segmented thalamic lesion masks were used to extract individual lesion volumes for further correlation analysis. The masks for deep cerebellar nuclei, dentate nucleus (DN), and interpositus nucleus (IN), were taken from the SPM neuroanatomy toolbox (51) and were imported in the CONN toolbox. All ROIs were thresholded to contain only voxels that were inside each ROI with a probability threshold above 60% (52). Notably, when extracting ROI-level BOLD signal, we opted to use the unsmoothed images to further avoid signal contamination from neighboring voxels of other proximal regions, which was especially important in using cerebellar ROIs that are very close to each other (53).

The ROI-to-ROI analyses consisted of the following steps. Each subject's first-level Fisher-Z transformed connectivity matrices (expressing pairwise correlations between the BOLD time series of each pair of ROIs) were subjected to a second-level within-group and within-subject analysis of variance, testing for FC differences across the three-time points (main contrast analysis, corresponding to the “*main effect of treatment”*: baseline vs. 1-mo vs. 3-mo). In this first analysis, the age and duration of disease were considered as a covariate of no interest to minimize their potential influences. Next, the disease duration, the 24-h individual lesion volume (mm^3^), the individual absolute drop points, as well as the % value of tremor improvement at 3-mo were separately fed into a regression model against the main contrast (baseline vs. 1-mo vs. 3-mo) to assess their impact on FC changes (respectively: “*treatment by disease duration*,” “*treatment by lesion volume*,” and “*treatment by clinical improvement*”–interaction effects). Then, a between-subject analysis comparing good vs. poor responders (GR vs. PR) was performed both across the three time points (“*treatment by outcome interaction effect*”), and only at baseline (“*pretherapeutic functional profiles of outcomes*”). All results were corrected at cluster-level by parametric multivariate statistics (cluster-level inferences, functional network connectivity-FNC) (54); with connection threshold set at *p* < 0.1 uncorrected, and cluster threshold set at *p* < 0.05 false discovery rate (FDR) corrected (multi-voxel pattern analysis omnibus test). Statistics outside the CONN toolbox were performed using the OriginPro 2015 (Origin Lab Corporation, Northampton, MA, USA).

## Results

### Final Sample Definition

Of the initial 60 patients with TD-PD, 20 subjects were found to be eligible for MRgFUS and were admitted to the fMRI longitudinal study. All patients successfully completed the MRgFUS Vim ablation. Four patients did not complete the rs-fMRI follow-up. One patient who completed the rs-fMRI follow-up was excluded from the group-analysis because of excessive head movement, thus, leaving 15 TD-PD in the final study sample. The demographics of the patients, including age, gender, disease duration, side of thalamotomy, and the 1-mo and 3-mo post-treatment tremor improvement for the treated body side relative to baseline, as well as 24-h lesion volumes, are summarized in Table 1.

**Table 1:** Demographic and clinical data of patients with Parkinson's disease with information on 1-mo and 3-mo post-treatment tremor improvement for the treated body side relative to baseline, as well as 24-h lesion volumes.

	**Age (yrs)**	**Sex**	**Disease duration (yrs)**	**Tx side**	**24 h**	**Tremor score for treated body side**	**Drop points (-)**	**Percentage (%) improvement**	**Drop points (-)**	**Percentage (%) improvement**
					**Lesion vol (mm^**3**^)**	At baseline^*^	**1-mo–baseline**	**At 1-mo**	**3-mo–baseline**	**At 3-mo**
PD 1	60	M	3	L	241	5	−3	60	−3	60
PD 2	71	M	19	R	280	5	−3	60	−4	80
PD 3	68	M	12	R	159	7	−3	43	−6	86
PD 4	77	M	10	L	456	7	−3	43	−6	86
PD 5	55	M	8	L	318	9	−6	67	−6	67
PD 6	63	M	4	R	337	6	−4	67	−3	50
PD 7	58	M	3	R	117	7	−5	71	−4	57
PD 8	61	F	5	R	230	7	−2	67	−2	67
PD 9	57	M	2	R	179	7	−1	14	−2	29
PD 10	68	M	19	L	380	11	−8	73	−4	36
PD 11	65	F	4	R	216	7	−2	29	−2	29
PD 12	61	M	1	L	392	6	−1	17	−2	33
PD 13	74	M	4	R	287	10	−4	40	−4	40
PD 14	53	M	4	R	231	10	0	0	0	0
PD 15	73	M	5	R	256	5	−2	40	−2	40
**Mean**	64		6.8		272	7.2	−3.1	46	−3.3	50.6
**(±SD; range)**	(±7; 53–77)		(±6; 1–19)		(±92.5; 117–456)	(±1.9; 5–11)	[±2.1; (-) 8–0]	(±23; 0–73)	[±1.7; (-) 6–0]	(±24.3; 0–86)

* *Scores are referred to tremor sub-items (3.15.a, 3.16.a, 3.17.a, and 3.17.c) of the MDS-UPDRS motor part in off-drug. Yrs, years; Tx, treated; vol, volume; M, male; F, female; L, left; R, right; 1-mo, 1 month after MRgFUS; 3-mo, 3 months after MRgFUS*.

By the 3-mo follow-up of neurological examination, the group of patients were divided based on tremor improvement in: PD-GR (*n* = 8), who differed significantly from PD-PR (*n* = 7) (*t* = 5.5, *p* < 0.001; 69.12 vs. 29.57%). The age, disease duration, and 24-h lesion volumes did not significantly differ between the (Good Responder) GR and the (Poor Responder) PR (see Table 2).

**Table 2:** Demographic and clinical data of GR- and PR- patients.

	**GR-PD**	**PR-PD**	**GR-PD vs. PR-PD (unpaired *t*-test)**
Age (ysr)	64.13 ± 7.34	64.43 ± 7.34	*t* = 0.0771 *P* = 0.9397
Sex (male/female)	7/1	6/1	
Disease duration (yrs)	8.00 ± 5.55	5.57 ± 6.08	*t* = 0.8097 *P* = 0.4333
TX side (L/R)	3/5	2/5	
24 h (lesion vol mm^3^)	267.25 ± 106.64	277.29 ± 81.48	*t* = 0.2023 *P* = 0.8428
Tremor score for treated body side (baseline)	6.63 ± 1.30	8.00 ± 2.31	*t* = 1.4462 *P* = 0.1718
Drop points (-): 1 mo–baseline	−3.63 ± 1.30	−2.57 ± 2.70	*t* = 0.9844 *P* = 0.3429
Percentage (%) improvement (1 mo)	59.75 ± 10.99	30.43 ± 23.71	***t*** **=** **3.1448** ***P*** **=** **0.0077**
Drop points (-): 3 mo – baseline	−4.25 ± 1.58	−2.29 ± 1.38	*t* = 2.5443 *P* = 0.0245
Percentage (%) improvement (3 mo)	69.13 ± 13.59	29.57 ± 13.82	***t*** **=** **5.5800** ***P*** **=** **0.0001**

*All values are expressed as Mean ± SD. significant results are highlighted in bold*.

### Main Effect of MRgFUS Treatment

**Figure 1:**
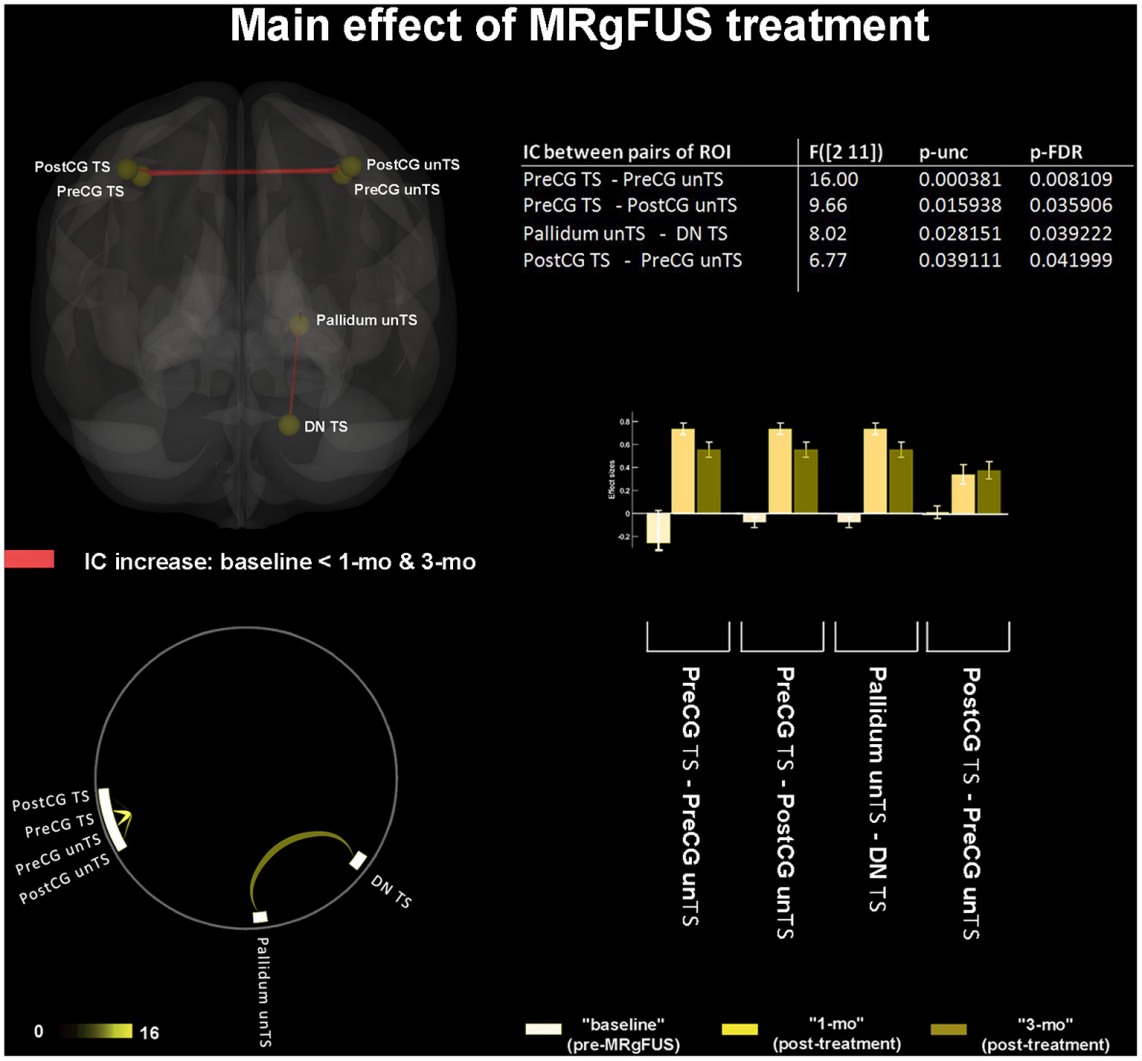
“Main effect” of MRgFUS treatment. The 3D brain rendering and circle connectome graph illustrate statistically significant results. Statistics between each pair of regions of interest (ROI) are detailed within the table and by the corresponding bar plot; IC, Interconnectivity.

A significant FC increase at 1-mo and 3-mo (as compared to baseline) was detected between: TS PreCG and unTS PreCG; TS PreCG and TS PostCG; unTS PreCG and TS PostCG; and unTS pallidum and unTS DN (Figure 1). No significant FC differences in any pair of connections were detected between post-treatment conditions (i.e., 1-mo vs. 3-mo).

### Treatment by Disease Duration Interaction Effect

**Figure 2:**
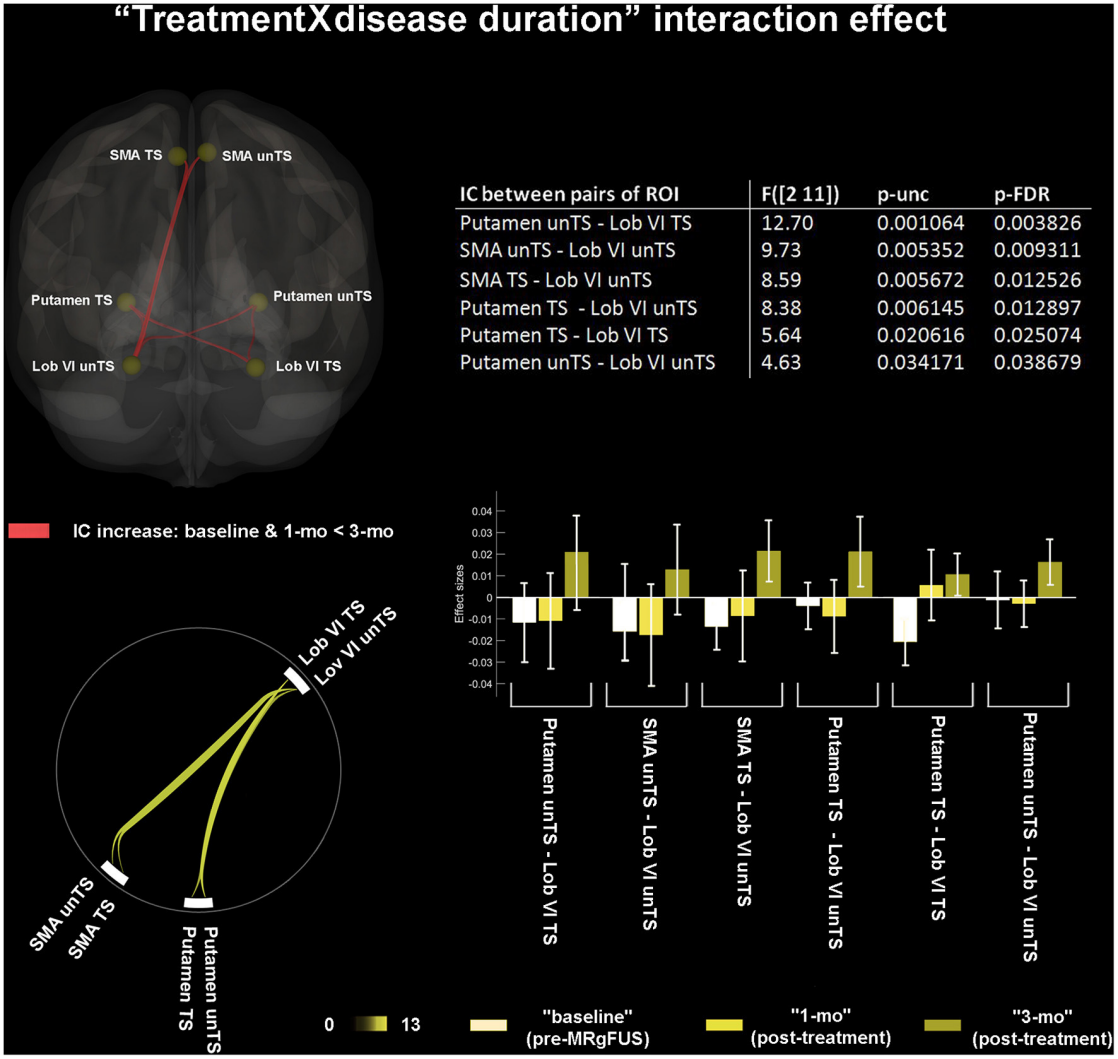
“Treatment by disease duration” interaction effect. 3D brain rendering and circle connectome graph illustrate statistical significant results of functional connectivity (FC) changes. Statistics between each pairs of ROI are detailed within the table and by the corresponding bar plot; IC, interconnectivity.

Significant correlations between disease duration and FC increase at 3-mo (as compared to baseline and 1-mo) were found between: unTS putamen and both TS, unTS Lob VI; TS putamen and both TS, unTS Lob VI; and both TS, unTS SMA and unTS Lob VI (Figure 2).

### Treatment by Lesion Volume Interaction Effect

No significant interaction effects were found between 24-h lesion volumes and post-treatment FC changes.

### Treatment by Clinical Improvement Interaction Effect

**Figure 3:**
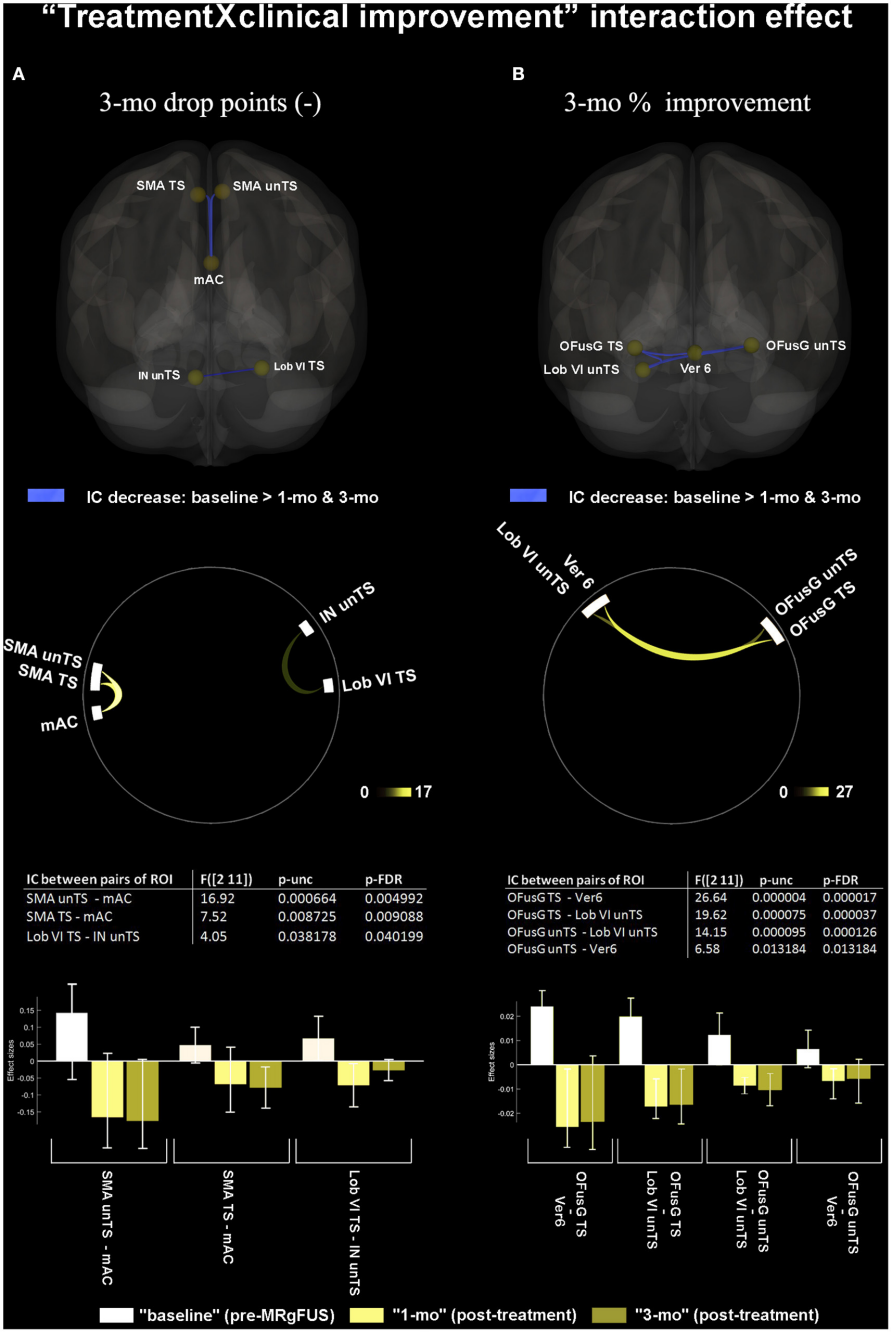
“Treatment by clinical improvement” interaction effect. **(A)** shows significant correlations between FC changes and tremor improvement at 3-mo, expressed as 3 months “absolute drop points” of MDS-UPDRS sub-items for tremor (3.15.a, 3.16.a, 3.17.a, and 3.17.c). **(B)** shows significant correlations between FC changes and tremor improvement at 3-mo, expressed as intra-subject “percentage of improvement.” The 3D brain rendering and circle connectome graph illustrate statistically significant results. Statistics between each pair of ROI are detailed within the table and by the corresponding bar plot; IC, Interconnectivity.

Significant correlations between the tremor improvement at 3-mo (expressed as drop score value) and the FC decrease in post-treatment (at 1-mo and 3-mo as compared to baseline) were found between mAC with TS, unTS SMA; and TS Lob VI and unTS IN (Figure 3A). Significant correlations between the tremor improvement at 3-mo (expressed as % intra-subject value) and the FC decrease in post-treatment (at 1-mo and 3-mo as compared to baseline) were found between: TS OFusG and unTS Lob VI, Ver VI; and unTS OFusG and unTS Lob VI, Ver VI (Figure 3B). When comparing post-treatment conditions (1-mo vs. 3-mo), no significant correlations were found between the measures of tremor improvement and the changes in FC.

### Treatment by Outcome Interaction Effect

**Figure 4:**
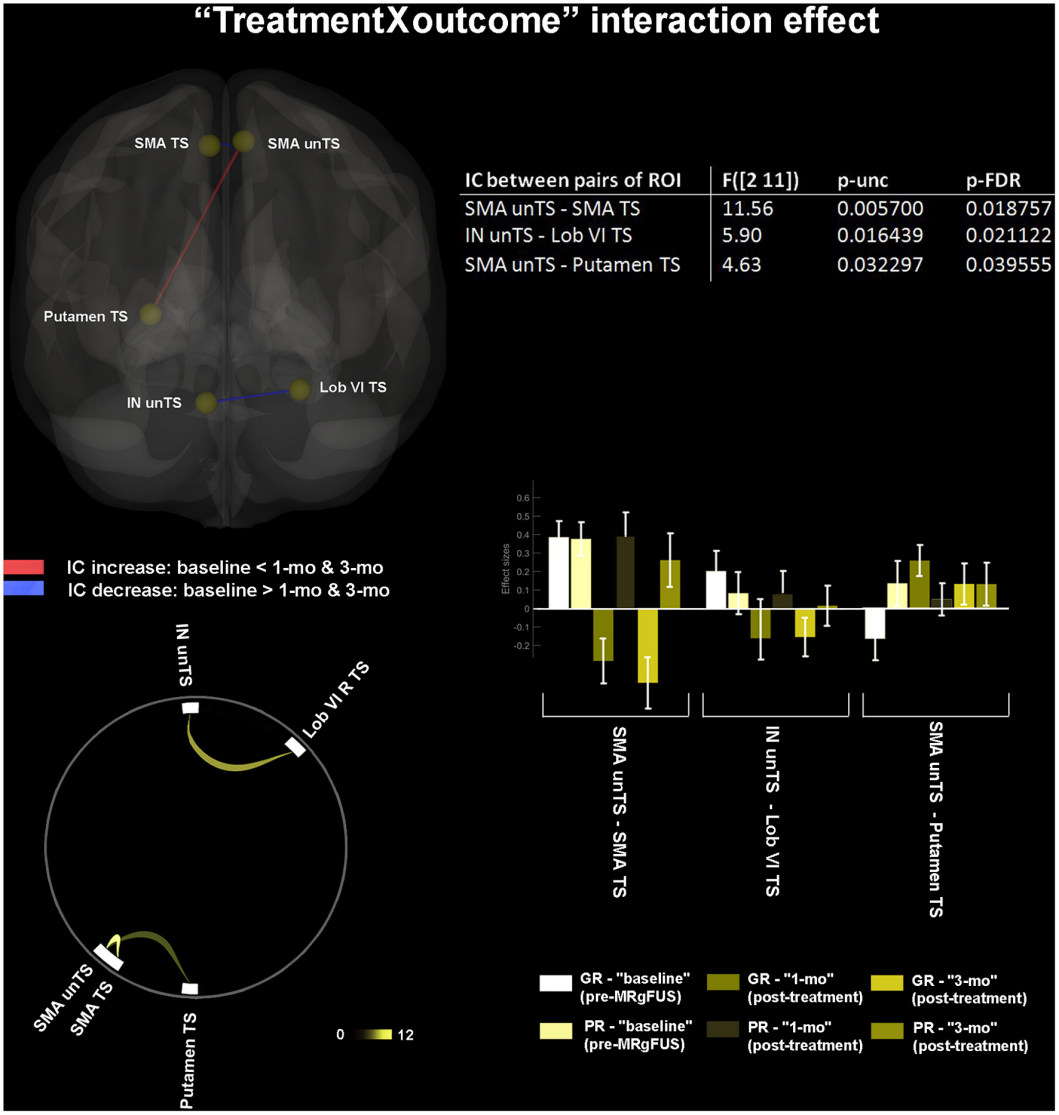
“Treatment by outcome” interaction effect. Within-subject longitudinal changes of FC selectively associated with the clinical outcome (good vs. poor response to treatment) are shown. The 3D brain rendering and circle connectomic graph illustrate statistically significant results. Statistics between each pair of ROI are detailed within the table and by the corresponding bar plot; IC, Interconnectivity.

Good Responder-Parkinson's Disease (GR-PD) showed a significantly reduced post-treatment FC (as compared to PR-PD) between: unTS and TS SMA; unTS IN and TS Lob VI. Conversely, they showed significantly increased FC between unTS SMA and TS putamen (Figure 4). The poor responders (PR) did not exhibit post-treatment FC changes in any pair of the ROIs connections.

### Pretherapeutic FC Profiles of Outcomes

**Figure 5:**
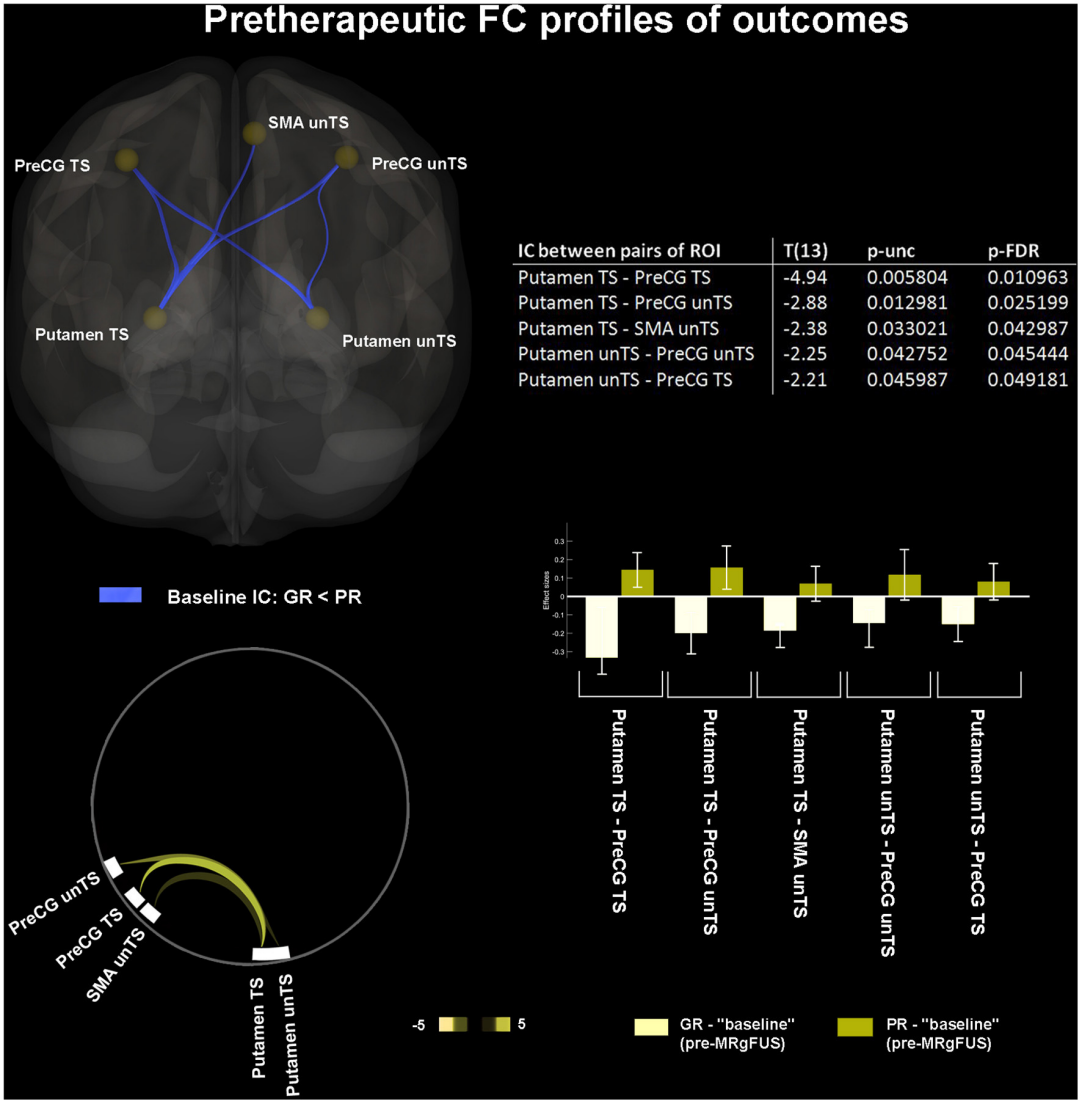
“Pretherapeutic functional profiles of outcomes.” Retrospectively identified baseline FC features associated with different clinical outcome (good vs. poor response to treatment). The 3D brain rendering and circle connectomic graph illustrate statistically significant results. Statistics between each pair of ROI are detailed within the table and by the corresponding bar plot; IC, Interconnectivity.

At baseline, the GR-PD showed significant hypoconnectivity (as compared to PR-PD) between: TS putamen and both TS and unTS PreCG; TS putamen and unTS SMA; and unTS putamen and both TS and unTS PreCG (Figure 5).

## Discussion

The Magnetic Resonance-guided high-intensity Focused Ultrasound (MRgFUS) is a new non-invasive neurosurgical procedure for improving parkinsonian tremor (55–57). It has been demonstrated to be safe and effective, at least not inferior to unilateral DBS (58), thus, providing clinicians with the choice for different options for a more appropriate intervention based on the features of the individual patients. The procedure is fully executed in the MRI setting, which allows real-time monitoring of the location and size of the lesion. Moreover, clinical effect, as well as any potential adverse event, can be promptly assessed. This aspect differentiates the MRgFUS from surgical lesional thalamotomy or radiotherapy. However, as the MRgFUS is a relatively recent technique, follow-up data and randomized clinical trials are quite limited (56).

There is only one report investigating the impact of MRgFUS Vim thalamotomy on the neuronal activity in a whole-brain level (42). In particular, the authors measured fractional amplitude of low-frequency fluctuations (fALFF) on nine medication-refractory of patients with TD-PD, finding significant changes in visual areas at 12 months after the treatment compared to baseline (42). On a different note, in our study, we assessed the effect on the brain FC of unilateral MRgFUS thalamotomy according to the commonly accepted pathogenic structure functional hypothesis of tremor, which is based on the cerebello-thalamo-cortical circuitries converging on the Vim. We conducted a hypothesis-driven ROI-to-ROI rs-fMRI analysis, exclusively focusing on tremor-related brain areas, to accomplish a “*single network”*-based description (59) of medium-term effects (i.e., at 1 and 3 months postoperatively) of the MRgFUS treatment. Therefore, this is the first study to explore the rs-FC changes after the MRgFUS selective thalamotomy adopting a “*classic”* ROI-based approach. Previous studies have investigated longitudinal MRgFUS modulation of both the *topological* brain networks properties and the *effective connectivity* by employing graph analysis (34) or spectral dynamic causal modeling (35) only in an ET population. Of note, all the previous works included have no more than 10 patients in their study sample, while we could achieve a larger sample of 15 TD-PD subjects.

We would like to emphasize that the investigation of the FC changes after MRgFUS for tremor offers a unique opportunity to identify the neural correlates of this symptom quite univocally, by dissociating it from the overall disease phenotype on a lesional (iatrogenic) basis. Although the MRgFUS effects are immediate, an extended time for the clinical follow-up has been arranged to observe the enduring FC changes associated with the sustained tremor relief, rather than with transient clinical effects that were potentially induced by vasogenic oedema surrounding the lesion (33).

We found that the rs-FC, between tremor-related brain areas, was effectively modulated by MRgFUS. Selective Vim lesion had remote effects, modifying the balance of FC between ROIs far from the site of the lesion. Therefore, we believe that the “whole” tremor-network should be considered as the ultimate target of MRgFUS thalamotomy in PD (59).

The FC increase between TS and unTS PreCG; TS PreCG and unTS PostCG; and unTS PreCG and TS PostCG was one of the main effects of the treatment. It may reflect interhemispheric reorganization within bilateral primary motor (M1) cortices, as well as between bilateral M1 with crossed primary somatosensory (S1) cortices, with a probable enhanced synchronicity in homotopic brain regions underlying coherent sensorimotor behavior. The importance of integrating and cooperating bilateral sensorimotor systems for appropriate motor performance has been highlighted in healthy subjects (60), as well as in post-stroke patients (61). However, the interhemispheric coordination in PD is still under investigation, with little shreds of evidence showing an inverse relationship between the degree of motor impairment and the functional coordination in sensorimotor regions (62), along with optimal interhemispheric neural synchronization of motor cortices after DBS (63). We found that tremor suppression after MRgFUS in patients with TD-PD was paralleled by a greater synchronization of intra-cortical sensorimotor functions. A remodulation of pathological cortico-strial and/or cortico-thalamic interactions caused by Vim ablation could explain this phenomenon. We could suppose that the MRgFUS thalamotomy was able to shift the system toward a more segregated functional state (64).

Another main effect of thalamotomy in PD was the increased FC between TS DN and unTS pallidum. Interactions between the cerebellum and the basal ganglia have been traditionally interpreted as indirectly occurring, *via* discrete multi-synaptic loops, primarily at the level of the cerebral cortex (65). However, recent research in primates using viral tracers has demonstrated bidirectional, disynaptic, and subcortical communication between the basal ganglia and the DN *via* the thalamus (66, 67). Our finding of an enhanced FC between an output stage of cerebellar processing (i.e., the DN), with an in-line station of basal ganglia processing (i.e., the pallidum), supports the existence of direct and reciprocal influences between these subcortical structures. Indeed, basal ganglia and cerebellum work synergistically to produce an efficient motor functioning, being both implicated in reinforcement learning, motor planning, and action understanding, as well as in sensorimotor prediction and control (68). Notwithstanding, the altered activity in cerebellar pathways has only recently been recognized as potentially important in PD tremorigenesis (69). The currently prevailing views emphasize that the cerebellar node of the tremor circuit (i.e., “the dimmer”) drives the tremor by manipulating its amplitude (11). Ma et al. (70) reported a higher dentato-cerebellar FC in TD-PD, interpreted as a compensatory mechanism overcoming the basal ganglia impairment, but ultimately favoring the tremor onset. By contrast, Liu et al. (71) found lower FC between the DN and the posterior cerebellum in TD-PD. Our finding of an increased dentate-pallidal FC, associated with a tremor relapse improvement after Vim thalamotomy, might suggest a pre-surgical thalamic interference between these two structures with increasing connectivity after treatment, according to Liu's hypothesis. Vim interference could result in their functional uncoupling, in terms of delay, asynchrony, or excessive local synchrony, causing a tremor-prone instability.

We also showed that the thalamotomy-induced FC increased between bilateral Lob VI of cerebellum with bilateral putamen and SMA. These effects correlated with a longer disease duration and were delayed, occurring only at 3-mo after the procedure. They were also distributed, involving both hemispheres regardless of treatment side. The Lob VI is associated with primary sensorimotor body representation in the cerebellum (72), has strong functional connections with premotor cortex (i.e. the SMA) (73), and plays a role in the temporal control of action sequences, as well as in sensorimotor processing of errors prediction (74). Functional impairment of the SMA is associated with the pathophysiology of PD, as it is directly implicated in motor planning (75). The SMA and the putamen are mutually connected and constitute the “readiness potential” of self-initiated actions, which is typically less prominent in PD (76). The post-treatment increase in functional synchrony between the SMA, putamen, and Lob VI would indicate a gain of function in this multicomponent cognitive-motor system, composed of discrete processes, occurring simultaneously, and aimed at effective motor performance.

The central role played by the altered patterns of FC, involving the SMA, putamen, and Lob VI, also emerged from other findings.

First, the clinical improvement on the treated body-side (expressed as 3-mo drop points at the MDS-UPDRS tremor sub items) was associated with decreased FC between bilateral SMA and mAC. The mAC is part of the so-called “cingulate motor areas” (77), which, in turn, belongs to the wider “supplementary motor complex” of the medial prefrontal cortex. Similar to the proximal pre-SMA, these areas contribute to second-order aspects of motor function (78–80).We couldn't topographically disentangle the involvement of the different cingulate motor areas along their rostro-caudal axis (77) due to inherent methodological limitations. This would have allowed a more accurate definition of the role of the mAC in the PD tremor. We can only suppose that Vim ablation induced functional reorganization within the supplementary motor complex, particularly between the anterior cingulate and the bilateral SMA, and that this effect, among others, best reflected the patient's clinical improvement. Speculatively, we could assume the presence of a previous aberrant functional recruitment among premotor areas of both the medial hemispheric was potentially related to tremor. However, we cannot definitively determine whether this mechanism was pathological in nature, or rather represented a maladaptive chronic process (8, 81).

Second, MRgFUS resulted in a significant increase of FC between unTS SMA and TS putamen only in good responders, who were also retrospectively characterized at baseline by reduced FC between these two areas–unlike poor responders who were not.

This pre-treatment hypoconnectivity pattern could be considered a potential FC predictor of MRgFUS response. Since the two prognostic sub-groups were not clinically different at baseline, such FC feature did not correspond to more severe symptoms. We could therefore hypothesize that either *clinical* and corresponding *functional* phenotypes matched quite inaccurately in our sample, or the observed *functional* feature reflected a greater susceptibility to thalamotomy efficacy. This latter hypothesis may rely on a greater predisposition to pathological functional decoupling between the SMA and the putamen. Perhaps, this predisposition may also occur on a structural basis, which needs to be addressed in future works. We should, however, emphasize that the proposed “decoupled” functional status of the SMA-putamen connection is an *indirect inference* of our “ablative iatrogenic study model.” In fact, the tremor relief after the FC increase between these two nodes does not necessarily demonstrate restoration of a specific circuit but could, eventually, simply implicate iatrogenic interference within a complex maladaptive loop on which the other remote masked amplifying mechanisms can chronically act upon. Hence, we cannot definitively determine whether the interaction between the two nodes works; *causing, sharing*, or simply *mediating* tremor mechanisms.

Nonetheless, our result underlines the importance of the “putamen-SMA” connection in the pathogenesis of TD-PD. Previous observations were quite inconsistent as to whether PD is characterized by stronger or by weaker putamen-SMA FC, compared with healthy subjects. For example, Wu et al. (82) reported a reduced FC, whereas Kwak et al. (83) and Yu et al. (84) reported an enhanced FC. Furthermore, none of these studies specifically accounted for tremor. The present findings support a critical role of putamen-SMA interaction in TD-PD by showing that a better response to treatment paralleled the reorganization of their connectivity, which consisted of an increased cross-functional coupling. In this context, the concomitant post-treatment decrease in inter-hemispheric connectivity between the SMA on both sides should be interpreted as a complementary regulation, perhaps, even reflecting reallocation of functional resources.

Third, good responders retrospectively exhibited limited pre-treatment connectivity between TS, unTS putamen, with both ipsilateral and contralateral PreCG. These results further corroborate the evidence that patterns of altered connectivity in the cortico-striatal loop in TD-PD primarily involves M1 (85, 86), the most critical area in motor execution. Notably, our results are in accordance with previous studies showing a reduced rs-FC between M1 and putamen in PD (87, 88). One might assume that such FC feature might correlate with the clinical picture of tremor before treatment. However, this feature did not correspond to more severe symptoms in our sample, since good and poor responders did not differ in tremor severity at baseline (see Table 2). We could, therefore, hypothesize that the *clinical* and the corresponding *functional* phenotypes do not always match accurately.

Fourth, we found that tremor improvement was also associated with post-treatment decrease of FC between the unTS Lob VI/Ver VI of cerebellum and the bilateral OFusG. These FC changes were correlated with clinical improvement of tremor. In line with Xiong et al. (42) we observed the involvement of the second-order, functionally highly-specialized, visual area in the pathogenesis of tremor in PD. Also, such contribution was already evidenced in ET (37, 40, 41, 89). Our finding of a reduced interaction between specific subareas of the occipital lobe and the cerebellar hemisphere supports the evidence that the remote influence between structurally segregated regions with distinct functional profiles may exist even in the absence of direct anatomical projections, through indirect polysynaptic pathways of connection (41, 90). Although the precise function of the OFusG has not been fully revealed yet, it has been implicated in high-level visual processing, such as categorical recognition of visual stimuli (91, 92), and in those processes characterized by high recurrence of perceptual ambiguity (93). It is noteworthy that the PD motor performance is prone to deterioration with increasing ambiguous visual stimuli. This may be due to the peculiar dysfunction of cerebellar forward models used to mitigate the effect of sensory uncertainty on motor performance (94), which would make patients with PD particularly sensitive to visual feedback (95). Therefore, a compensatory pre-treatment increase of FC between the OFusG and the Lob VI/Ver VI of cerebellum–areas that are preferentially activated in the visual guidance of complex limb movement (96)–could be plausible. Conversely, the fact that the greater tremor relief paralleled the reduced FC between these areas would suggest an adaptive and reversible nature of this functional coupling.

Finally, a reduction in good responders between TS Lob VI and unTS IN was observed, following MRgFUS. This effect correlated with clinical improvement (expressed as absolute value of drop in MDS-UPDRS III sum score for contralateral tremor sub items). This finding supports the hypothesis that the tremor in PD would be associated with an increased activity within the cerebellum (97). The IN is part of the olivo-cortico-nuclear kinematic microcircuit, which is responsible for appropriate timing signals for movement coordination during ongoing motor performance. It also participates in the development of internal models for dynamic motor regulation in response to the external environment (98). The finding of the reduced FC between Lob VI and IN in patients who relieved more would suggest possible remodulation of intra-cerebellar functional resources associated with effective treatment.

### Limitations

Some limitations need to be mentioned. First, the small sample size (*n* = 15) may have limited statistical power to identify a less robust effect. This could eventually explain the absence of results in poor responders. Otherwise, it could suggest that ineffective treatments did not determine appreciable FC changes, as well as that characteristic pre-therapeutic profiles might not be recognizable in poor responders. We look forward to multi-center studies sharing data from advanced imaging techniques, which would allow for a wider patient recruitment and longer follow-up with, consequently, more robust results.

Second, although patients were always examined in a pharmacological washout, we cannot rule out the prolonged effect of chronic therapy on brain FC (99). It would be advisable for future studies to explore potential FC changes induced by Vim thalamotomy in PD, while controlling for “off” and “on” states. We are currently proceeding toward this purpose.

Third, the interhemispheric connections *via* the corpus callosum explain quite exhaustively the “crossed” pattern of many of our results between cerebral hemispheres. However, the presence of “uncrossed” functional interactions between supratentorial structures and cerebellum might be not immediately justifiable. We suggest that they may be explained either anatomically–by the presence of the non-decussating cerebellar pathways (100)–or functionally–by the intrinsic nature of endogenous BOLD signal fluctuations, which reflect the spontaneous correlation between distant brain regions as long as they are, somehow, functionally related (101).

Fourth, the rs-FC is a correlational technique, expressing temporal synchrony among BOLD fluctuations at rest between different couple of ROIs. Our analysis is solely correlational. Therefore, we did not provide information about the directed causal influences among involved brain regions (the so-called “effective connectivity”), nor could rule out moderation-mediation effects due to third parts (the so-called “partial correlation” analysis). We can interpret our network-based description of FC changes following MRgFUS only in terms of re-modulation and spatial re-allocation of functional resources. Moreover, we cannot definitively determine if these effects were reactive rather than causative, nor if they corresponded to the restoration of a “*normal old function”* or to a “*treatment specific signature”* superimposed on maladaptive adjustments of a chronically disrupted system.

Last, we found no association between the 24-h MRgFUS lesion volume and clinically relevant post-treatment FC changes. Previous radiological studies on MRgFUS (mainly based on morphological data) suffer from some inconsistency, with most authors reporting fewer symptom recurrences with larger lesions (46, 102–106), while others were focusing more on lesion location (20, 107) or topography (27, 108–110), rather than the lesion volume. Indeed, we observed some heterogeneity in the size and shape of Vim lesions in our sample, whereas lesion volumes did not differ significantly between GR and PR. Therefore, the absence of a correlation between FC changes and the lesion volumes did not particularly surprise us. Future studies on larger samples of patients need to investigate potential interference of lesion volume on FC, which may not have emerged in our study.

### Conclusions

We demonstrated for the first time with a ROI-to-ROI connectomic approach how MRgFUS VIM thalamotomy modulates rs-FC of the tremor network in patients with TD-PD. We showed that treatment-mediated changes of FC between specific sub-regions of this diffuse network correlated with the tremor clinical improvement. Taken together, our results demonstrated a shifting mode of cooperation among the constituents of the tremor network that is susceptible to external redirection despite the chronic nature of disease. Finally, we identified the pre-surgical FC interactions that are potentially associated with greater tremor improvement after thalamotomy, suggesting their possible “predictive” use. Future studies in larger samples of PD subjects are mandatory to validate the utility of rs-FC as a quantifiable biomarker of tremor improvement after MRgFUS.

## Data Availability Statement

The datasets presented in this article are not readily available because the files cannot be deposited in an accessible repository online in compliance with the regulations on the processing and dissemination of personal data in the health sector (application of the EU Regulation 2016/679 (GDPR) and the Privacy Italian Law as updated by Legislative Decree 101/2018). Requests to access the datasets should be directed to the corresponding author.

## Ethics Statement

The study was reviewed and approved by Scientific Direction-Ethics Committee Fondazione IRCCS Istituto Neurologico Carlo Besta (protocol n°74). The patients/participants provided their written informed consent to participate in this study.

## Author Contributions

MS conceived the study, executed MRgFUS procedures as neuroradiologist, collected and referred radiological data, analyzed rs-fMRI data and drew inferences, and wrote the first and latest version of the manuscript. NG and SB carried out the neurological assessment, supported the selection of patients for intervention, executed MRgFUS procedures as neurologist, monitored clinical outcomes, and contributed to the interpretation of the results. SR carried out the neurophysiopathological examination, assisted MRgFUS procedures, and defined organizational and procedural aspects of patients' selection for intervention. SP performed descriptive statistics, contributed to the interpretation of the results, and wrote the second and current version of the manuscript. AN contributed to the interpretation of the results. FG, GD, and JM pre-processed and segmented anatomical data. GM and GT executed MRgFUS as neurosurgeons, monitored clinical outcomes, and contributed to the interpretation of the results. LD'I collected and referred radiological data, executed MRgFUS procedures as neuroradiologist, and contributed to the interpretation of the results. FDM contributed to the conception of the study and to the interpretation of the results. RE contributed to the conception of the study, carried out the neurological assessment, supported the selection of patients for intervention, executed MRgFUS procedures as neurologist, monitored clinical outcomes, contributed to the interpretation of the results, and supervised all stages of work. MGB contributed to the conception of the study, executed MRgFUS procedures as neuroradiologist, collected and referred radiological data, and supervised all stages of work. All authors contributed to the article and approved the submitted version.

## Conflict of Interest

GF was employed by company InSightec Ltd. The remaining authors declare that the research was conducted in the absence of any commercial or financial relationships that could be construed as a potential conflict of interest.

## Publisher's Note

All claims expressed in this article are solely those of the authors and do not necessarily represent those of their affiliated organizations, or those of the publisher, the editors and the reviewers. Any product that may be evaluated in this article, or claim that may be made by its manufacturer, is not guaranteed or endorsed by the publisher.

## Abbreviations

BOLD: Blood oxygenation level dependent
CSF: cerebrospinal fluid
dPMC: dorsal premotor cortex
DN: dentate nucleus
DRTT: dentato-rubro-thalamic tract
FC: functional connectivity
FDR: false discovery rate
GM: gray matter
GR: good responder
IN: interpositus nucleus
Lob: lobule
mAC: median anterior cingulate
MDS-UPDRS: Movement Disorder Society Unified Parkinson's Disease Rating Scale
MNI: Montreal Neurological Institute
MRgFUS: Magnetic Resonance-guided high-intensity Focused Ultrasound
OFusG: occipital fusiform gyrus
PaCiG: paracingulate gyrus
PR: poor responder
PreCG: precentral gyrus
PostCG: postcentral gyrus
ROI: region-of-interest
rs-fMRI: Resting state functional MRI
T: tesla
SDR: skull density ratio
SPL: superior parietal lobe
SMA: supplementary motor area
SPM: the Statistical Parametric Mapping
STN: the subthalamic nucleus
TD-PD: tremor-dominant Parkinson's disease
Th: thalamus
TOFusG: temporal-occipital fusiform cortex
TS: treated side
unTS: untreated side
Ver: cerebellum–vermis subdivisions
Vim: thalamic ventral intermediate nucleus
vPMC: ventral premotor cortex
WM: white matter.
